# Plasma serine and glycine in relation to clinical outcomes following neoadjuvant radiotherapy for rectal cancer

**DOI:** 10.1016/j.ctro.2026.101159

**Published:** 2026-04-06

**Authors:** Nienke R.K. Zwart, Per Magne Ueland, Adrian McCann, Jill A. McKay, Heidi Rütten, Johannes A. Bogers, Johannes H.W. de Wilt, Ellen Kampman, Dieuwertje E. Kok

**Affiliations:** a Division of Human Nutrition and Health, Wageningen University & Research, Wageningen, the Netherlands; b BEVITAL AS Laboratory, Bergen, Norway; c School of Geography and Natural Sciences, Northumbria University, Newcastle upon Tyne, UK; d Department of Radiation Oncology, Radboud University Medical Center, Nijmegen, the Netherlands; e Radiotherapy Group, Arnhem, the Netherlands; f Department of Surgery, Radboud University Medical Center, Nijmegen, the Netherlands

**Keywords:** Rectal cancer, Serine, Glycine, Radiotherapy, Cancer recurrence

## Abstract

**Introduction:**

There is substantial variation in radiotherapy response among patients with rectal cancer. The amino acids serine and glycine have been proposed as radiosensitizers in preclinical studies. Here, we explored associations between plasma serine, glycine, and their ratio and clinical outcomes following neoadjuvant treatment for rectal cancer, including tumour downstaging and cancer recurrence.

**Methods:**

Based on a prospective cohort study, 288 patients with stage I-III rectal cancer were included. Blood was collected around diagnosis and plasma levels of serine and glycine were measured using GC–MS/MS. Tumour downstaging was defined as T-classification downstaging (pT < cT) after neoadjuvant treatment and recurrence included locoregional recurrences and distant metastases occurring in the 5-years after surgery. Regression models were used to calculate relative risks (RR) and hazard ratios (HR) and their 95% confidence intervals (CI) for tumour downstaging and recurrence, respectively.

**Results:**

Tumour downstaging was observed in 41% (n = 117) of the patients and the 5-year recurrence rate was 23% (n = 67). Serine, glycine, and their ratio were not associated with tumour downstaging. A higher serine/glycine ratio was associated with a lower risk of cancer recurrence (HR_perdoubling_ 0.47, 95%CI 0.22–0.99) and tumour downstaging was associated with lower risk of cancer recurrence (HR 0.40, 95%CI 0.22–0.70).

**Conclusion:**

This is the first study demonstrating a potential association between the serine/glycine ratio, an indicator of SHMT enzyme activity, and rectal cancer recurrence. Further studies are warranted to confirm these findings, as well as investigate underlying biological mechanisms and potentially explore strategies to target SHMT enzyme activity in the context of cancer treatment.

## Highlights

• Radiotherapy response varies across patients with rectal cancer.
• Preclinical studies propose the amino acids serine and glycine as radiosensitizers.
• Plasma serine and glycine were measured prospectively in 288 patients with rectal cancer.
• Plasma serine and glycine were not associated with tumour downstaging.
• Higher serine/glycine ratio was associated with a lower risk of cancer recurrence.

## Introduction

Radiotherapy is a common treatment applied modality for multiple types of cancer, including rectal cancer [1]. About two-thirds of all patients with rectal cancer receive neoadjuvant radiotherapy [2], [3], either as short-course radiotherapy alone or as radiotherapy with concurrent chemotherapy (chemoradiation) [4], [5]. There is notable variation in response to radiotherapy and chemoradiation, with studies reporting pathological complete response in ∼ 5–12% [6], [7], [8] and ∼ 15–27% [7], [8], [9] of the patients with rectal cancer, respectively. So far, underlying mechanisms for interindividual differences in radiotherapy response remain unclear and the ability to predict tumour downstaging is limited [10], [11], [12].

Based on a preclinical study, it has been suggested that restriction of the amino acids serine and glycine may cause radiosensitization and hence improve response to radiotherapy in human colorectal cancer cell lines and patient-derived rectal cancer organoids [13]. Serine and glycine are involved in DNA repair and redox responses, which is hypothesized to explain why their depletion may result in better response to radiotherapy [14]. Related to this, the enzyme serine hydroxymethyltransferase 2 (SHMT2), which is responsible for the conversion of serine to glycine in mitochondria [15], has been observed to be highly expressed in radiation-resistant gastric cancer cell lines and depletion of SHMT2 resulted in radiosensitization [16]. Inhibition of SHMT2 combined with radiotherapy further reduced lung tumour growth in mice compared to receiving radiotherapy alone [17]. These findings suggest that lower SHMT activity, and hence a higher serine/glycine ratio [18], may be associated with more favourable responses to radiotherapy and, therefore, cancer prognosis.

Despite compelling findings in preclinical studies, the precise role of plasma serine and glycine in patients with rectal cancer remains unknown. Hence, the aim of this study was to explore the association between plasma serine, glycine, and the serine/glycine ratio with clinical outcomes, including tumour downstaging after neoadjuvant treatment and cancer recurrence, in patients with rectal cancer.

## Materials and methods

### Study population

Data from the ‘COlorectal cancer: Longitudinal, Observational study on Nutritional and lifestyle factors that influence colorectal tumour cancer recurrence, survival and quality of life’ (COLON) study (NCT03191110, ClinicalTrials.gov) were used. Adults newly diagnosed with colorectal cancer were recruited from 11 hospitals in The Netherlands between 2010–2020. Details have been described previously [19]. The COLON study was approved by a medical ethics committee (region Arnhem-Nijmegen, 2009–349). All participants provided written informed consent.

**Fig. 1:**
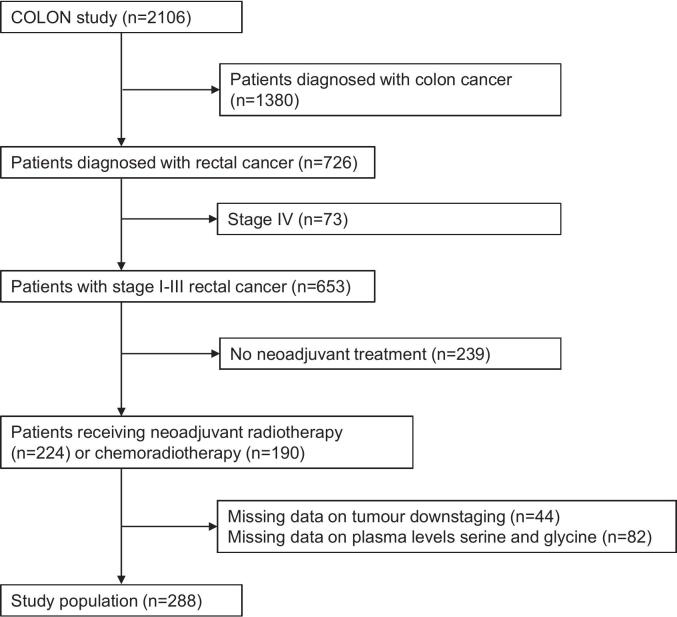
Flow chart showing the selection of the study population.

Among the 2106 patients included in the COLON study, 726 patients were diagnosed with rectal or rectosigmoid cancer (Fig. 1). Patients diagnosed with metastatic cancer (n = 73) were excluded. Patients with rectal cancer were eligible for the current study if they received neoadjuvant radiotherapy or chemoradiation (n = 414). A total of 126 patients were excluded due to missing tumour staging data (n = 44) or blood samples (n = 82), resulting in a study population of 288 patients.

### Serine and glycine

Non-fasting blood samples were collected in EDTA tubes either before (n = 168, median (IQR) 11 (6–20) days) or after (n = 112, median (IQR) 40 (7–91) days, n = 8 unknown) start of neoadjuvant treatment. There appeared to be no difference in serine and glycine concentrations collected before or after start treatment (Supplementary File 1). Samples were centrifuged at 1300xg at 4 °C for 15 min and plasma was extracted and stored at −80 °C. Serine (µmol/L) and glycine (µmol/L) were measured using isotope-labelled internal standards and gas chromatography-tandem mass spectrometry (GC–MS/MS) [20] by BEVITAL AS (www.bevital.no), Norway. The within- and between-day coefficient of variation (CV) for both serine and glycine ranged from 1 to 2%. In the same samples, pyridoxal 5′-phosphate (PLP) (nmol/L) was measured using liquid chromatography-tandem mass spectrometry (LC-MS/MS) [21], as PLP is a cofactor of SHMT [22]. Within- and between-day CV for PLP were 4% and 6%, respectively. The serine/glycine ratio was calculated as indicator for SHMT activity [23], [24]. Higher serine/glycine ratios indicate higher concentrations of substrate (serine) relative to product (glycine) and therefore suggest decreased SHMT activity [18].

### Outcomes

#### Tumour downstaging

Tumour downstaging was based on the clinical tumour (cT1-4) classification, determined at diagnosis and before start of neoadjuvant treatment, and the pathological tumour (pT1-4) classification, determined after neoadjuvant treatment and surgery. The cT classifications for rectal carcinoma are commonly determined by MRI scan in the Netherlands [25]. All patients in this study underwent surgery, largely in line with national treatment protocols operating at that time [5] and inherent to recruitment strategies of the COLON study. Tumour downstaging was defined as a decrease in tumour size measured as pT < cT [26], [27], [28]. Classifications T1 and T2 were grouped together as it is challenging to distinguish between T1 and T2 based on imaging techniques [29]. TNM classifications were derived through the Netherlands Cancer Registry via the Netherlands Comprehensive Cancer Organization (IKNL).

#### Cancer recurrence

Recurrence data were obtained through IKNL, most recently updated in July 2022. Recurrence was defined as local or regional cancer recurrences, or distant metastases detected in the 5-years after surgery (5-years recurrence). Follow-up time was defined as time from date of surgery until date of recurrence, death, loss of follow-up, or the cut-ff point of 5-years after surgery, whichever came first. If date of surgery was unknown (n = 3), follow-up time was calculated based on the date of blood drawn.

### Covariates

At diagnosis, patients filled out a semi-quantitative food questionnaire (FFQ) of 204 items to assess habitual alcohol assumption the month prior to diagnosis (g/day) [30], [31] and a general questionnaire including questions about age at inclusion (years), sex (women/men), body weight (kg), height (cm), and smoking status (former/current/never). Data on clinical characteristics, such as tumour location and type of neoadjuvant treatment, were collected via IKNL and complemented with data from the Dutch ColoRectal Audit (DCRA) [32] and medical records in case of missings.

### Data analysis

Data were presented as median with interquartile range (IQR, Q1-Q3) or numbers with percentages. For analyses with tumour downstaging as outcome, Cox proportional hazards regression analyses with a constant time variable were used to calculate relative risks (RR) and 95% confidence intervals (CI) using the Huber-White estimator [33]. RRs are here preferred over odds ratios as the outcome is common [34]. For analyses with recurrence as outcome of interest, Cox proportional hazards regression models were used to calculate hazard ratios (HRs) and 95%CIs. Patients with missing recurrence data were excluded from this analysis (n = 1). Plasma marker concentrations and serine/glycine ratio were log2-transformed. Risk estimates should therefore be interpreted per doubling in plasma marker or ratio. Crude models were adjusted for age and sex. Based on literature [35], [36], [37], [38], [39], the following covariates were also evaluated: tumour size at diagnosis (cT1 + 2/3/4), alcohol intake, body mass index (BMI, kg/m^2^), smoking status, and type of neoadjuvant treatment (radiotherapy/chemoradiation). Additionally, for analyses with the serine/glycine ratio as exposure, PLP concentrations (nmol/L) were evaluated as potential confounder [22]. Covariates were added one by one to the respective models. If covariates changed the RR/HR with > 10%, the covariate was added. The fully adjusted model for tumour downstaging included sex, age, tumour size at diagnosis, smoking status, and type of neoadjuvant treatment. The fully adjusted model for cancer recurrence included age, sex, tumour size at diagnosis, and type of neoadjuvant treatment.

A stratified analysis was performed based on type of neoadjuvant treatment (radiotherapy (n = 155) or chemoradiation (n = 133)). Also, a sensitivity analysis was performed excluding patients with blood samples collected after start neoadjuvant treatment (n = 112) or unknown time of blood collection (n = 8). Data analyses were performed with R Statistical Software (version 4.3.2). Statistical significance was defined as 95%CI not including 1.

## Results

Patients had a median (IQR) age of 64 (58–70) years at diagnosis and 33% were women (Table 1). More patients were diagnosed with stage III rectal cancer (73%) compared to stage I and II (9% and 18%, respectively). Radiotherapy alone (54%), in the Netherlands usually prescribed as short course radiotherapy (5x5Gy), was more often prescribed than chemoradiation (46%) (usually as 25x2Gy with capecitabine) [5], [25]. Compared to those receiving chemoradiation, the patients receiving radiotherapy were more often men (71% versus 62%), had more often cT1-2 classifications (32% versus 9%), less often pT0 classifications (2% versus 22%), and less time between end of neoadjuvant treatment and surgery date (2 versus 70 days) (Supplementary File 2).

**Table 1:** Characteristics of the study population by clinical outcomes following neoadjuvant treatment.

		Tumour downstaging		Cancer recurrence^5^	
	Total study population (n = 288)	Yes (n = 117, 41%)	No (n = 171, 59%)	Yes (n = 67, 23%)	No (n = 220, 76%)
**Demographic and lifestyle factors**					
Women / men	95 (33%) / 193 (67%)	46 (39%) /71 (61%)	49 (29%) / 122 (71%)	25 (37%) / 42 (63%)	70 (32%) / 150 (68%)
Age (years)	64 [58–70]	63 [57–69]	64 [58–70]	63 [57–70]	64 [58–70]
BMI (kg/m^2^)^1^	25.9 [24.2–28.7]	25.8 [23.6–29.4]	25.9 [24.3–28.0]	25.9 [24.3–27.8]	25.9 [24.1–28.9]
Smoking status^2^					
Current	34 (12%)	18 (16%)	16 (10%)	6 (10%)	28 (13%)
Former	163 (58%)	60 (54%)	103 (61%)	33 (53%)	130 (60%)
Never	82 (30%)	33 (30%)	49 (29%)	23 (37%)	58 (27%)
Alcohol intake (g/day)^3^	9.0 [0.9–20.4]	8.0 [0.7–17.4]	9.6 [1.2–22.5]	9.0 [0.4–18.7]	9.3 [1.6–21.0]
Neoadjuvant treatment					
Radiotherapy	155 (54%)	46 (39%)	109 (64%)	30 (45%)	124 (56%)
Chemoradiation	133 (46%)	71 (61%)	62 (36%)	37 (55%)	96 (44%)
Surgery	288 (100%)	117 (100%)	171 (100%)	67 (100%)	220 (100%)
Time between end neoadjuvant treatment and surgery date (days)^4^	44 [2–74]	58 [5–81]	5 [2–68]	64 [4–83]	32 [2–68]
**Disease staging before neoadjuvant treatment**					
cT-classification					
T1-2	62 (22%)	6 (5%)	56 (33%)	8 (12%)	53 (24%)
T3	203 (71%)	90 (77%)	113 (66%)	51 (76%)	152 (69%)
T4	23 (8%)	21 (18%)	2 (1%)	8 (12%)	15 (7%)
cStage^5^					
I	25 (9%)	3 (2%)	22 (13%)	4 (6%)	20 (9%)
II	53 (18%)	21 (18%)	32 (19%)	10 (15%)	43 (20%)
III	209 (73%)	93 (80%)	116 (68%)	53 (79%)	156 (71%)
**Disease staging after neoadjuvant treatment**					
pT-classification					
T0	32 (11%)	32 (27%)	0 (0%)	4 (6%)	28 (13%)
T1-2	111 (39%)	71 (61%)	40 (23%)	12 (18%)	99 (45%)
T3	138 (48%)	14 (12%)	124 (73%)	48 (72%)	89 (41%)
T4	7 (2%)	0 (0%)	7 (4%)	3 (5%)	4 (2%)
pStage^5^					
0	29 (10%)	29 (25%)	0 (0%)	3 (5%)	26 (12%)
I	82 (29%)	55 (47%)	27 (16%)	8 (12%)	74 (34%)
II	71 (25%)	6 (5%)	65 (38%)	19 (28%)	52 (24%)
III	106 (37%)	27 (23%)	79 (46%)	37 (55%)	68 (31%)
Tumour downstaging	117 (41%)	117 (100%)	0 (0%)	20 (30%)	97 (44%)
5-year cancer recurrence	67 (23%)	20 (17%)	47 (28%)	67 (100%)	0 (0%)
**Plasma markers**					
Serine concentrations (µmol/L)	99.4 [86.0–114.0]	100.0 [85.0–117.0]	99.2 [87.2–112.0]	97.4 [86.8–111.0]	99.9 [86.0–116.0]
Glycine concentrations (µmol/L)	224 [189–261]	226 [192–276]	221 [187–255]	226 [191–265]	222 [189–260]
Serine/glycine ratio	0.44 [0.37–0.52]	0.44 [0.37–0.52]	0.45 [0.38–0.51]	0.41 [0.35–0.49]	0.45 [0.38–0.52]
PLP concentrations (nmol/L)	42.2 [33.1–59.5]	42.4 [34.5–61.8]	42.0 [31.3–59.0]	42.2 [29.0–60.1]	42.1 [33.6–59.5]
Data missing for ^1^ 2 patients, ^2^ 9 patients, ^3^ 10 patients, ^4^ 21 patients, ^5^ 1 patient.					

**Fig. 2:**
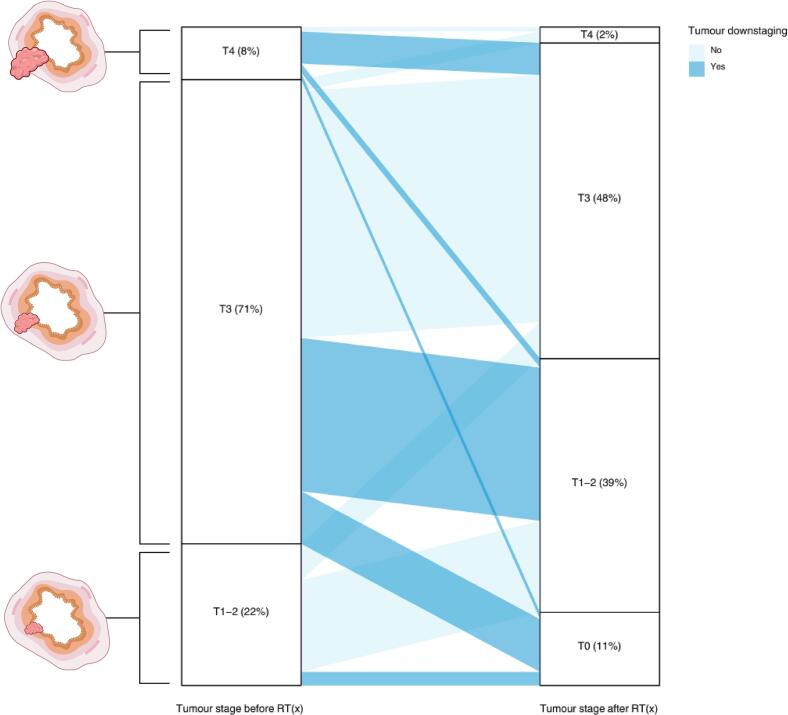
Overview of the change in tumour stage before and after neoadjuvant treatment in the study population. Created with BioRender.com. Abbreviations: RT(x) neoadjuvant radiotherapy or chemoradiation.

A total of 117 (41%) patients had tumour downstaging after neoadjuvant treatment (Fig. 2). Compared to those without tumour downstaging, the patients with downstaging were more often women (39% versus 29%), diagnosed with stage III rectal cancer (80% versus 68%), more often received chemoradiation (61% versus 36%), and had a longer time between completion of neoadjuvant treatment and surgery (58 days versus 5 days). During the follow-up of 5.0 (IQR 2.9–5.0) years, a total of 67 (23%) rectal cancer recurrences were observed after surgery. The patients with a recurrence more often had stage III after neoadjuvant treatment (55% versus 31%), more often received chemoradiation (55% versus 44%), and had less tumour downstaging (30% versus 44%) as compared to patients without recurrent disease (Table 1).

### Tumour downstaging and cancer recurrence

Plasma concentrations of serine, glycine, and the serine/glycine ratio were not statistically significantly associated with tumour downstaging (Table 2). The stratified analyses by neoadjuvant treatment and the sensitivity analyses excluding patients with blood sample collection after start of neoadjuvant treatment or with unknown collection date showed similar results (Supplementary File 3–4).

**Table 2:** Associations of plasma concentrations of serine, glycine, and serine/glycine ratio with tumour downstaging after neoadjuvant treatment.

Plasma markers	Model 1			Model 2		
	n/events	RR	95% CI	n/events	RR	95% CI
Serine, per doubling	288/117	1.35	0.78–2.34	279/111	1.37	0.76–2.46
Glycine, per doubling	288/117	1.25	0.75–2.09	279/111	0.99	0.57–1.73
Serine/glycine ratio, per doubling	288/117	1.05	0.60–1.83	279/111	1.34	0.78–2.29
Model 1: Adjusted for age (in years) and sex (women, men) Model 2: Adjusted for age (in years), sex (women, men), tumour size at diagnosis (cT1 + 2, 3, 4), smoking status (current, former, never), and neoadjuvant treatment (radiotherapy, chemoradiation)						

A higher serine/glycine ratio was associated with a lower risk of 5-year cancer recurrence (HR_perdoubling_ 0.47, 95% CI 0.22–0.99) (Table 3). This association was specifically seen in patients receiving radiotherapy (HR_perdoubling_ 0.21, 95% CI 0.07–0.60) but not in those receiving chemoradiation (HR_perdoubling_ 0.84, 95% CI 0.29–2.37) (Supplementary File 3). Serine and glycine separately were not statistically significantly associated with risk of 5-year cancer recurrence in the overall population. However, higher glycine concentrations were associated with a higher risk of cancer recurrence in patients receiving radiotherapy (HR_perdoubling_ 2.98, 95% CI 1.07–8.35) but not chemoradiation (HR_perdoubling_ 0.92, 95% CI 0.33–2.59) (Supplementary File 3). The associations did not markedly change after excluding patients with blood sample collection after start of neoadjuvant treatment or with unknown collection date (Supplementary File 4). Patients with tumour downstaging after neoadjuvant treatment had a lower risk of 5-year cancer recurrence compared to patients with no tumour downstaging (HR 0.40, 95% CI 0.22–0.70) (Table 3).

**Table 3:** Associations of plasma concentrations of serine, glycine, the serine/glycine ratio, or tumour downstaging with 5-year cancer recurrence.

	Model 1			Model 2		
	n/events	HR	95% CI	n/events	HR	95% CI
Plasma markers						
Serine, per doubling	287/67	0.66	0.32–1.35	287/67	0.66	0.32–1.38
Glycine, per doubling	287/67	1.36	0.68–2.70	287/67	1.36	0.67–2.77
Serine/glycine ratio, per doubling	287/67	**0.45**	**0.22**–**0.94**	287/67	**0.47**	**0.22**–**0.99**
Tumour downstaging						
No tumour downstaging	170/47	Ref		170/47	Ref	
Tumour downstaging	117/20	**0.58**	**0.34**–**0.98**	117/20	**0.40**	**0.22**–**0.70**
Model 1: Adjusted for age (in years) and sex (women, men) Model 2: Adjusted for age (in years), sex (women, men), tumour size at diagnosis (cT1 + 2, 3, 4), and neoadjuvant treatment (radiotherapy, chemoradiation)						

## Discussion

In this study, we investigated plasma serine, glycine, and the serine/glycine ratio in relation to clinical outcomes after neoadjuvant treatment, including tumour downstaging and cancer recurrence, in a prospective cohort of 288 patients with non-metastatic rectal cancer. Serine, glycine, and the serine/glycine ratio were not associated with tumour downstaging. However, a higher serine/glycine ratio appeared to be associated with a lower risk of cancer recurrence.

The serine/glycine ratio is considered an indicator for SHMT enzyme activity [18], [23], [24] and is widely used in this context in clinical studies largely focussing on mental health [23], [24], [40], [41], [42]. Reduced SHMT activity and hence a higher serine/glycine ratio, may result in decreased production of the antioxidant glutathione, NADPH, and purines [43], [44], which has been hypothesized to favour treatment response and ultimately cancer prognosis [14]. While previous preclinical studies suggested that knockdown or pharmacological inhibition of SHMT2 might result in radiosensitization and hence improve responses to radiotherapy [16], [17], we did not observe an association between the serine/glycine ratio and tumour downstaging. We did, however, observe an association between a higher serine/glycine ratio, indicating lower SHMT activity, and a lower risk of cancer recurrence. These findings suggest that SHMT activity may play a role in cancer prognosis. In a *meta*-analysis of 10 studies including a total of 1,952 patients with various types of cancer, including colorectal cancer, higher expression of SHMT2 was associated with worse prognosis (either progression-free survival, disease-free survival, or recurrence-free survival: HR 1.90, 95% CI 1.31–2.76) [45]. Building on the assumption that the serine/glycine ratio is a proxy for SHMT activity, this finding is in line with our explorative results. Altogether, these results may highlight the potential importance of SHMT activity in cancer prognosis. The serine/glycine ratio may be considered a good proxy for SHMT activity, especially within the context of large cohort studies for which measuring enzyme activity is not always feasible.

To the best of our knowledge, this is the first clinical study investigating circulating concentrations of serine and glycine separately, and the serine/glycine ratio, in relation to tumour downstaging in patients with rectal cancer. As indicated earlier, some previous findings have suggested a role of serine/glycine metabolism in radiotherapy response in cancer cell lines and tumour-derived rectal cancer organoids [13], [15], [16]. One previous prospective study investigated the role of pretreatment tumour glycine concentrations in relation to tumour downstaging after chemoradiation as well as progression-free survival in 54 patients with rectal cancer [46]. Similar to our study, no statistically significant association was observed between glycine and tumour downstaging measured as tumour regression grade (no hazard ratio provided). However, the study did observe that higher tumour glycine concentrations were associated with poorer progression-free survival (HR 4.4 95% CI 1.4–14.3). The authors hypothesized that glycine concentrations might reflect tumour aggressiveness, particularly high proliferation rates, as well as tumour hypoxia [46]. In our explorative study, we observed an association between higher plasma glycine and a lower serine/glycine ratio and higher risk of cancer recurrence in patients receiving radiotherapy but not chemoradiation. The role of metabolism may be limited in the patients receiving chemoradiation, as their prognostic signature was worse compared to those receiving radiotherapy (more often diagnosed with cT3-4 and clinical stage III), therefore other tumour characteristics, such as residing cancer cells and tumour dormancy, may be more important as drivers of recurrence risk [47]. To what extent other patient or treatment-related factors could explain potential differences for treatments types remains unclear. Also, it should be noted that the sample sizes for the subgroups based on type of neoadjuvant treatment were modest, possibly resulting in insufficient power to detect associations. Whether and how plasma glycine is related to cancer prognosis, either via a direct interaction with radiotherapy or as a marker of cancer aggressiveness, warrants further research.

The current study has some limitations. First, in clinical studies primarily designed to evaluate radiotherapy response as main outcome, the RECIST criteria and tumour regression grade (TRG) are commonly used instead of T-classification downstaging [48], [49], [50]. In our study, these criteria could not be applied, as required data were not available. Clinical staging, as used in our study, might be prone to underestimation of the tumour size [51]. However, as tumour downstaging was strongly associated with a lower risk of cancer recurrence in our study, we regard it as a proxy for radiotherapy response, although limited to measures of primary tumour size and it should be acknowledged that also patients without tumour downstaging could exhibit a radiotherapy response. Second, although our study population is of modest size, albeit comparable or larger than that of earlier studies on radiotherapy responses [46], [52], [53], [54], power to demonstrate associations might be limited, especially when studying subgroups based on treatment strategies or local recurrences versus distant metastasis. Also, ongoing studies conducted at the time of the COLON study (such as RAPIDO [55]) might have resulted in some variation in treatment regimens. Third, the blood samples were collected in non-fasting conditions, making serine and glycine concentrations potentially susceptible to recent dietary intake [56], although previous studies observed minimal (<10%) differences between fasted and non-fasted serine and glycine concentrations [57], [58]. Moreover, the non-fasting conditions may have caused variation within the data but are unlikely to result in overestimation of the observed associations as these variations are most likely independent of the outcome of interest. Last, full details on dosing, fractionation or timing of treatments have not been uniformly registered and not all blood samples were collected before the start of neoadjuvant treatment, which could have resulted in altered plasma marker concentrations due to the treatments. In our study, however, there seemed to be no major difference between plasma concentrations of serine and glycine in samples collected before or after the start of neoadjuvant treatment. Moreover, the sensitivity analyses in which patients with samples collected after the start of neoadjuvant treatment were excluded showed similar findings as compared to the main analyses. The strengths of this study include assessment of serine, glycine, and serine/glycine ratio separately in a well-defined population with relevant clinical and lifestyle data available. To the best of our knowledge, this is the first study investigating serine, glycine, and the serine/glycine ratio in relation to clinically relevant outcomes after neoadjuvant treatment for rectal cancer.

In conclusion, we observed no association between plasma concentrations of serine, glycine, and serine/glycine ratio and tumour downstaging after neoadjuvant treatment among patients with non-metastatic rectal cancer. A higher serine/glycine ratio, however, was associated with a lower risk of cancer recurrence. Further studies are warranted to confirm these explorative findings and potentially explore strategies to target SHMT enzyme activity in the context of cancer treatment.

## Data availability statement

Data are available upon reasonable request. Since the data consist of identifying cohort information, some access restrictions apply and therefore cannot be made publicly available. Data will be shared with permission from the steering committee of the COLON study. Requests for data can be sent to dr. Dieuwertje Kok, Division of Human Nutrition and Health, Wageningen University & Research, The Netherlands (dieuwertje.kok@wur.nl).

## CRediT authorship contribution statement

**Nienke R.K. Zwart:** Conceptualization, Formal analysis, Investigation, Methodology, Project administration, Visualization, Writing – original draft. **Per Magne Ueland:** Resources, Writing – review & editing. **Adrian McCann:** Resources, Writing – review & editing. **Jill A. McKay:** Conceptualization, Writing – review & editing. **Heidi Rütten:** Methodology, Resources, Writing – review & editing. **Johannes A. Bogers:** Methodology, Resources, Writing – review & editing. **Johannes H.W. de Wilt:** Methodology, Resources, Writing – review & editing. **Ellen Kampman:** Conceptualization, Funding acquisition, Methodology, Project administration, Resources, Supervision, Writing – review & editing. **Dieuwertje E. Kok:** Conceptualization, Funding acquisition, Investigation, Methodology, Project administration, Resources, Supervision, Visualization, Writing – review & editing.

## Funding

Funding for grant number IIG_Full_2021_023 was obtained from Wereld Kanker Onderzoek Fonds (WKOF) as part of the World Cancer Research Fund International grant programme. The COLON study was financially supported by Wereld Kanker Onderzoek Fonds (WKOF) & World Cancer Research Fund International (WCRF International) as well as by funding (2014/1179, IIG_FULL_2021_022, IIG_FULL_2021_023, and IIG_FULL_2023_017) obtained from Wereld Kanker Onderzoek Fonds (WKOF) as part of the World Cancer Research Fund International grant programme; Alpe d’Huzes/Dutch Cancer Society (UM 2012–5653, UW 2013–5927, UW 2015–7946); ERA-NET on Translational Cancer Research (TRANSCAN via the Dutch Cancer Society (UW2013-6397, UW2014-6877) and the Netherlands Organization for Health Research and Development (ZonMw), the Netherlands); the Regio Deal Foodvalley (162135); and the Dutch Research Council (KICH1.LG01.22.009).

## Declaration of Competing Interest

The authors declare that they have no known competing financial interests or personal relationships that could have appeared to influence the work reported in this paper.
