# Supplementary material for: Plasma serine and glycine in relation to clinical outcomes following neoadjuvant radiotherapy for rectal cancer
Source: Clin Transl Radiat Oncol. 2026 Apr 6;59:101159. doi: 10.1016/j.ctro.2026.101159 (PMC13091465; doi:10.1016/j.ctro.2026.101159)
Supplement: Supplementary Data 1 [file mmc1.docx]

**
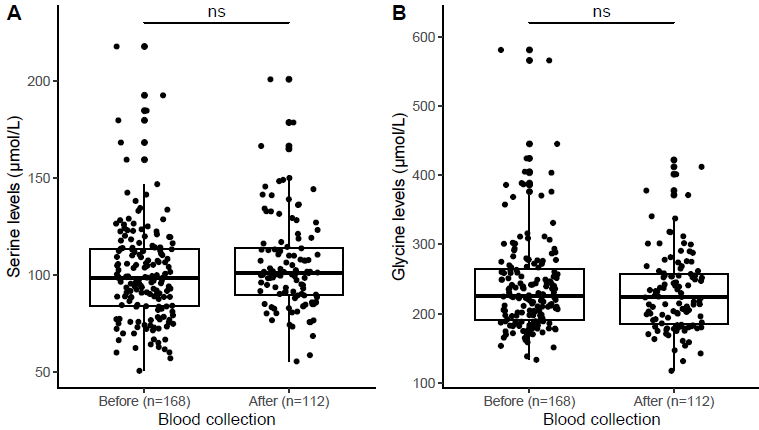
**

**Supplementary File 1.** A) Serine concentrations by timing of blood collection (before or after the start of neoadjuvant treatment), B) Glycine concentrations by timing of blood collection (before or after the start of neoadjuvant treatment). Patients with unknown blood collection date (n=8) were excluded from the graphs. Box plots represent the median and the inter-quartile range (IQR, Q1-Q3). P-values were calculated by Mann Whitney U tests; P-values<0.05 were deemed statistically significant. Abbreviation: ns not significant.

**Supplementary File 2.** Characteristics of the study population stratified by neoadjuvant treatment (radiotherapy and chemoradiation).

|  | Total study population (n=288) | Radiotherapy (n=155, 54%) | Chemoradiation  (n=133, 46%) |
| --- | --- | --- | --- |
| **Demographic and lifestyle factors** | | | |
| Women / men | 95 (33%) / 193 (67%) | 45 (29%) / 110 (71%) | 50 (38%) / 83 (62%) |
| Age (Years) | 64 [58-70] | 65 [60-73] | 63 [56-67] |
| BMI (kg/m^2^) ^1^ | 25.9 [24.2-28.7] | 26.0 [24.3-28.7] | 25.9 [24.2-28.8] |
| Smoking status ^2^ |  |  |  |
| Current | 34 (12%) | 13 (9%) | 21 (17%) |
| Former | 163 (58%) | 98 (65%) | 65 (51%) |
| Never | 82 (29%) | 41 (27%) | 41 (32%) |
| Alcohol intake (g/d) ^3^ | 9.0 [0.9-20.4] | 9.7 [1.6-22.2] | 7.9 [0.5-18.4] |
| **Disease staging before neoadjuvant treatment** | | | |
| cT |  |  |  |
| 1-2 | 62 (22%) | 50 (32%) | 12 (9%) |
| 3 | 203 (71%) | 97 (63%) | 106 (80%) |
| 4 | 23 (8%) | 8 (5%) | 15 (11%) |
| cStage ^4^ |  |  |  |
| I | 25 (9%) | 20 (13%) | 5 (4%) |
| II | 53 (18%) | 34 (22%) | 19 (14%) |
| III | 209 (73%) | 100 (65%) | 109 (82%) |
| **Disease staging after neoadjuvant treatment** | | | |
| pT |  |  |  |
| 0 | 32 (11%) | 3 (2%) | 29 (22%) |
| 1-2 | 111 (39%) | 73 (47%) | 38 (29%) |
| 3 | 138 (48%) | 77 (50%) | 61 (46%) |
| 4 | 7 (2%) | 2 (1%) | 5 (4%) |
| pStage |  |  |  |
| 0 | 29 (10%) | 2 (1%) | 27 (20%) |
| I | 82 (29%) | 51 (33%) | 31 (23%) |
| II | 71 (25%) | 34 (22%) | 37 (28%) |
| III | 106 (37%) | 68 (44%) | 38 (29%) |
| Tumour Downstaging | 117 (41%) | 46 (30%) | 71 (53%) |
| Time between end neoadjuvant treatment and surgery date (days) ^5^ | 44 (2-74) | 2 (1-5) | 70 (58-83) |
| Recurrence ^6^ | 67 (23%) | 30 (20%) | 37 (28%) |
| **Plasma markers** | | | |
| Serine (µmol/L) | 99.4 [86.0–114.0] | 98.8 [83.9–112.0] | 100 [90.3-117.0] |
| Glycine (µmol/L) | 224 [189-261] | 220 [186-254] | 230 [192-269] |
| Serine/glycine ratio | 0.443 [0.37-0.52] | 0.452 [0.38-0.52] | 0.436 [0.37-0.52] |
| PLP (nmol/L) | 42.2 [33.1-59.5] | 42.8 [33.1-62.0] | 40.8 [33.1-57.4] |
| Data missing for ^1^ 2 patients, ^2^ 9 patients, ^3^ 10 patients, ^4^ 1 patient, ^5^ 21 patients. ^6^ Cancer recurrence occurring in the 5 years after surgery. | | | |

**Supplementary File 3.** Associations of plasma concentrations of serine, glycine, and the serine/glycine ratio and tumour downstaging after neoadjuvant radiotherapy with 5-year cancer recurrence, stratified by neoadjuvant treatment (radiotherapy and chemoradiation).

|  | Radiotherapy | | | Chemoradiation | | |
| --- | --- | --- | --- | --- | --- | --- |
| Tumour downstaging^1^ | | | | | | |
|  | n/events | RR | 95% CI | n/events | RR | 95% CI |
| Serine, per doubling | 152/44 | 1.66 | 0.72-3.80 | 127/67 | 1.26 | 0.63-2.54 |
| Glycine, per doubling | 152/44 | 1.04 | 0.47-2.30 | 127/67 | 0.90 | 0.44-1.83 |
| Serine/glycine ratio, per doubling | 152/44 | 1.57 | 0.72-3.46 | 127/67 | 1.43 | 0.69-2.95 |
| Cancer recurrence^2^ | | | | | | |
|  | n/events | HR | 95% CI | n/events | HR | 95% CI |
| Serine, per doubling | 154/30 | 0.60 | 0.20-1.80 | 133/37 | 0.77 | 0.28-2.17 |
| Glycine, per doubling | 154/30 | **2.98** | **1.07-8.35** | 133/37 | 0.92 | 0.33-2.59 |
| Serine/glycine ratio, per doubling | 154/30 | **0.21** | **0.07-0.60** | 133/37 | 0.84 | 0.29-2.37 |
| No tumour downstaging | 108/24 | Ref |  | 62/23 | Ref |  |
| Tumour downstaging | 46/6 | **0.23** | **0.07-0.83** | 71/14 | 0.49 | 0.24-1.01 |
| ^1^ Adjusted for age (in years), sex (women, men), tumour size at diagnosis (cT1+2, 3, 4), smoking status (current, former, never)  ^2^ Adjusted for age (in years), sex (women, men), tumour size at diagnosis (cT1+2, 3, 4) | | | | | | |

**Supplementary File 4.** Associations of plasma concentrations of serine, glycine, and the serine/glycine ratio with tumour downstaging after neoadjuvant treatment and with cancer recurrence, excluding the patients with blood samples collected after start of neoadjuvant treatment (n=112) and unknown blood sample collection date (n=8).

|  | Model 2 in main manuscript | | | Model 2 - Only pretreatment samples | | |
| --- | --- | --- | --- | --- | --- | --- |
| Tumour downstaging^1^ | | | | | | |
|  | n/events | RR | 95% CI | n/events | RR | 95% CI |
| Serine, per doubling in µmol/L | 288/117 | 1.37 | 0.76-2.46 | 164/63 | 1.85 | 0.82-4.15 |
| Glycine, per doubling in µmol/L | 288/117 | 0.99 | 0.57-1.73 | 164/63 | 1.31 | 0.65-2.67 |
| Serine/glycine ratio, per doubling | 288/117 | 1.34 | 0.78-2.29 | 164/63 | 1.28 | 0.66-2.46 |
| Cancer recurrence^2^ | | | | | | |
|  | n/events | HR | 95% CI | n/events | HR | 95% CI |
| Serine, per doubling in µmol/L | 287/67 | 0.66 | 0.32-1.36 | 168/42 | 0.49 | 0.19-1.23 |
| Glycine, per doubling in µmol/L | 287/67 | 1.36 | 0.68-2.71 | 168/42 | 1.39 | 0.57-3.40 |
| Serine/glycine ratio, per doubling | 287/67 | **0.45** | **0.21-0.94** | 168/42 | **0.34** | **0.13-0.85** |
| ^1^ Adjusted for age (in years), sex (women, men), tumour size at diagnosis (cT1+2, 3, 4), smoking status (current, former, never), and neoadjuvant treatment (radiotherapy, chemoradiation)  ^2^ Adjusted for age (in years), sex (women, men), tumour size at diagnosis (cT1+2, 3, 4), and neoadjuvant treatment (radiotherapy, chemoradiation) | | | | | | |
